# Oocytes express an endogenous red fluorescent protein in a stony coral, *Euphyllia ancora*: a potential involvement in coral oogenesis

**DOI:** 10.1038/srep25868

**Published:** 2016-05-11

**Authors:** Shinya Shikina, Yi-Ling Chiu, Yi-Jou Chung, Chieh-Jhen Chen, Yan-Horn Lee, Ching-Fong Chang

**Affiliations:** 1 Institute of Marine Environment and Ecology, National Taiwan Ocean University, Keelung, 20224, Taiwan; 2 Center of Excellence for the Oceans, National Taiwan Ocean University, Keelung, 20224, Taiwan; 3 Department of Aquaculture, National Taiwan Ocean University, Keelung, 20224, Taiwan; 4 Institute of Oceanography, National Taiwan University, Taipei, 10617, Taiwan; 5 Tungkang Biotechnology Research Center, Fisheries Research Institute, Tungkang, 20246, Taiwan

## Abstract

To date,the molecular and cellular mechanisms underlying coral sexual reproduction remain largely unknown. We then performed a differential screen to identify genes related to oogenesis in the stony coral *Euphyllia ancora*. We identified a clone encoding a novel *red fluorescent protein* cDNA of *E. ancora* (named *EaRFP*). Microscopic observation and quantitative RT-PCR revealed that EaRFP is almost exclusively expressed in the ovary of the adult coral. The combination of the ovarian-cell separation method and the RT-PCR analysis revealed that the oocytes, but not the ovarian somatic cells, are the cells expressing EaRFP. Immunohistochemical analysis revealed that the expression of EaRFP starts in the early stage of the oocyte and continues until the maturation period. Furthermore, recombinant EaRFP was shown to possess an H_2_O_2_ degradation activity. These results raise the possibility that EaRFP plays a role in protecting the oocytes from oxidative stress from the early to late stages of oogenesis. The present study provides not only the first evidence for the potential involvement of FPs in coral oogenesis but also an insight into a cellular strategy underlying coral sexual reproduction.

Stony corals are indispensable in building the framework of coral reefs, which possess the highest levels of marine biodiversity on earth^1^. Sexual reproduction of corals is among the most important means of establishing new coral colonies, populations, and hence coral reefs. In sexual reproduction, the gametes or larvae released into the ocean allow corals to colonize different habitats and create genetic links between one reef region and another^2^. Sexual reproduction also allows corals to foster genetic diversity, an advantage in adapting to environmental changes.

Gametogenesis is the initiation of sexual reproduction and creates mature gametes that contain all the material to drive fertilization and embryonic development. To date, coral gametogenesis has been investigated in many species and locations worldwide mainly from ecological and histological perspectives^3^,^4^,^5^. The common characteristics of gametogenesis (e.g., processes, specific site of gametogenesis within the polyp, and the cycle of gametogenesis) among species have been revealed^3^,^4^,^5^. Generally, the annual spawning pattern of corals exhibits a single annual gametogenic cycle that is completed in less than 12 months^3^,^4^. Corals develop their gametes in a specific site of each endodermal mesentery^3^,^4^. Although cnidarians (e.g. corals, sea anemones, hydras, and jellyfish) are generally thought to possess no true organs, the specific site of gamete production is often referred to as a gonad (testis/ovary)^4^. Oogenesis begins with the proliferation of oogonia alongside the gonadal mesoglea, which is a gelatinous extracellular matrix. After the oogonia differentiate into early oocytes by entering meiosis, the oocytes enlarge in size and infiltrate into the gonadal mesoglea. Consequently, the oocytes are enveloped by a thin mesogleal layer in which they increase in size by accumulating yolk until they reach the maturation period^3^. On the other hand, spermatogenesis begins with spermatogonial proliferation alongside the gonadal mesoglea^6^,^7^. After spermatogonia form clusters, they infiltrate into the gonadal mesoglea and form spermaries that consist of dozens of spermatogonia surrounded by a thin layer of mesoglea. Spermatogonial proliferation, meiotic differentiation, and spermiogenesis take place within each spermary^3^,^6^,^7^.

Recently, several studies on coral gametogenesis from the molecular and cellular perspectives have been performed, and molecules related to coral gametogenesis have been identified^8^,^9^,^10^,^11^,^12^. Furthermore, the research bases required for detailed studies, such as a genomic database^13^ and molecular markers for germ cells^6^,^7^, have gradually been developed. However, the current understanding of the mechanisms underlying coral gametogenesis is quite limited. Because of increasing human concerns about reef degradation, the significance of coral reef conservation and coral biology have become topics of general interest. Therefore, further investigation of coral reproduction at the molecular and cellular levels would provide valuable information not only for the field of comparative biology but also for applied biology, such as the artificial induction of gametogenesis and spawning under aquaculture conditions.

A differential screen (suppression subtractive hybridization [SSH]) is one of the reliable methodologies to identify genes that are differentially expressed between two samples, such as between a non-reproductive sample and a reproductive sample^14^. Previously, we reported a study describing the SSH-based identification and characterization of a novel yolk protein cDNA, named *euphy*, in a gonochoric stony coral, *Euphyllia ancora*^15^. It was demonstrated that the euphy protein is synthesized by ovarian somatic cells, transported to, and abundantly accumulated in oocytes during oogenesis^15^.

Through the course of our SSH screening to explore oogenesis-related genes, we identified several clones that were up-regulated during the reproductive period of the female *E. ancora*. Preliminary homology searches in GenBank using BLAST (NCBI) showed that one of the clones, #2-6, exhibited similarities to the green fluorescent proteins of other corals. Because to date, the biological relationship between fluorescent proteins (FPs) and oogenesis has been relatively unexplored in corals, this prompted us to perform the further characterization of the clone. To the best of our knowledge, there are only a few studies that describe the results of microscopic observation of FPs in the released eggs of two coral species, *Pocillopora verrucosa*^16^ and *Montipora capitata*^17^,^18^. Here, we present the molecular identification and functional characterization of a novel red fluorescent protein that is endogenously, and almost exclusively, expressed in the oocytes from an early stage of oogenesis in the adult *E. ancora*.

## Results

### Suppression subtractive hybridization (SSH), molecular cloning, cDNA sequence determination and analysis

**Figure 1:**
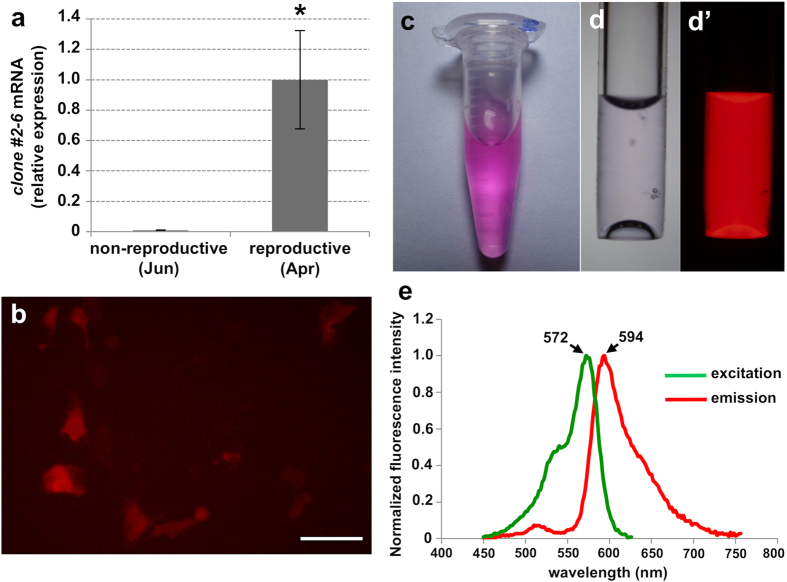
Molecular characterization of clone #2-6 (EaRFP) identified by a differential screen. (**a**) Comparison of transcript levels of clone *#2-6* (*EaRFP*) between non-reproductive (June) or reproductive (April) female colonies assessed by quantitative RT-PCR analysis. Data are shown as means ± SE (n = 4 colonies) relative to the non-reproductive female colonies (June). Significant differences were found between groups (Student *t*-test, *P* < 0.05). (**b**) Human embryonic kidney (HEK 293) cells expressing clone #2-6 protein (EaRFP). Scale bar = 20 μm. (**c**) Purified protein solution of recombinant clone #2-6 (EaRFP). (**d**) Micrograph of purified protein solution of recombinant clone #2-6 (EaRFP) in a glass capillary, bright view (**d**) and fluorescent view (**d**′). (**e**) Excitation and emission spectra of clone #2-6 (EaRFP).

Quantitative RT-PCR analysis revealed that the expression levels of a clone *#2-6*, which was identified by the screening of SSH cDNA library, were significantly up-regulated during the female reproductive period (April, 2011) compared to the non-reproductive period (June, 2010) in the female reproductive tissues which encompass the ovary (Fig. 1a). The first homology search of GenBank using BLAST (NCBI) showed that clone #2-6 exhibited similarity to the green fluorescent protein (GFP)-like protein. For the further characterization, we obtained the full-length cDNA sequence. The full-length cDNA was 1,079 bp in length and contained an open reading frame (ORF) of 678 bp corresponding to 226 amino acid residues, a 5′ untranslated region (UTR) of 85 bp, and a 3′ UTR of 321 bp including a poly A tail. The predicted molecular mass was 25.5 kDa. The second homology search of GenBank by BLAST using the obtained full-length cDNA sequence showed that the #2-6 exhibited the highest similarity to the chromo-red fluorescent GFP-like protein of the stony coral *Echinopora forskaliana* (GenBank accession No. ACD13196). The deduced amino acid sequence contained the tripeptide xYG, which is the chromophore motif typical of GFP family members (Supplementary Fig. 1a). Analysis of the phylogenetic relationship among the red fluorescent protein family members showed that clone #2-6 clustered with the chromo-red fluorescent GFP-like protein from the coral *E. forskaliana* (Supplementary Fig. 1b).

### Molecular characterization of clone #2-6

To further characterize clone *#2-6*, we used recombinant protein expression systems. First, HEK293 cells were transfected with an expression vector containing the ORF of clone #2-6. Fluorescent microscopic observation revealed that the transfected cells expressing #2-6 exhibited red fluorescence under a U-MWIG2 filter, which is used for the detection of the red fluorescent protein (Fig. 1b). No other fluorescence, such as green or cyan, was observed when the transfected cells were excited by other microscopic filters with different wavelengths. Additionally, no other fluorescence was observed in the cells transfected with the empty vector (control). Second, to investigate the emission and excitation spectra of clone #2-6, we expressed an N-terminal His-tagged recombinant clone #2-6 in *E. coli*. The purified recombinant #2-6 solution exhibited a visible clear pink color (Fig. 1c), and presented an intense red fluorescence under excitation by the 520–550 nm wavelengths with a U-MWIG2 filter (Fig. 1d,d’). Analysis of the spectrum revealed that clone #2-6 possesses an excitation_max_ of 572 nm and an emission_max_ of 594 nm (Fig. 1e), demonstrating that #2-6 is a red fluorescent protein. Based on these results, we tentatively named clone #2-6 as *E. ancora* red fluorescent protein (EaRFP; GenBank accession No. KT452623).

### Identification of EaRFP expressing tissue

**Figure 2:**
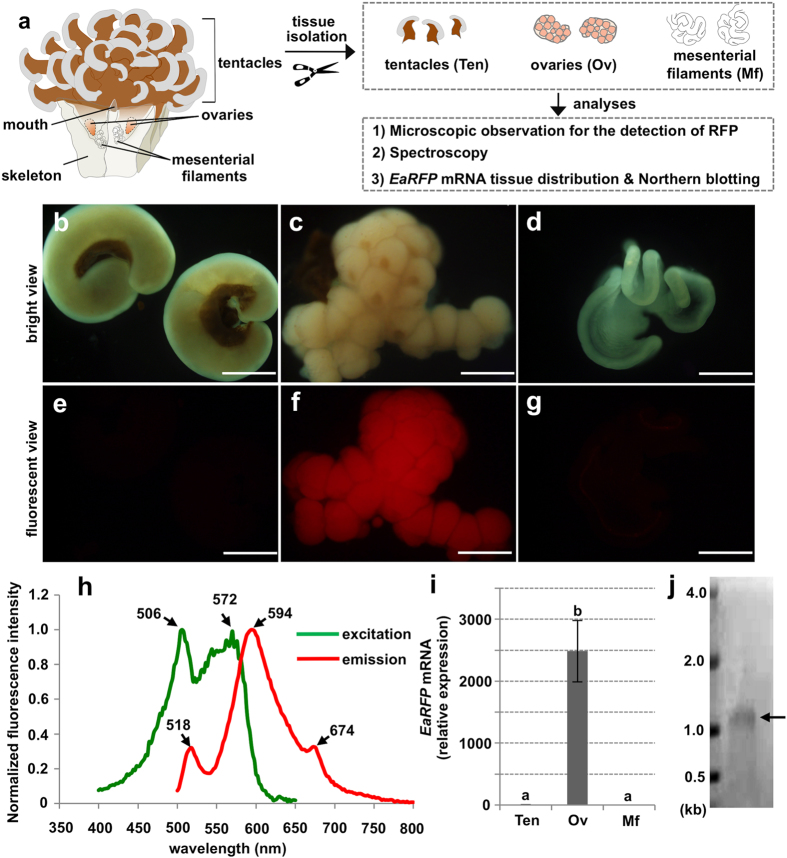
Identifiation of EaRFP expressing tissue in *E. ancora* polyp. (**a**) Schematic of *E. ancora* body structure (left panel), and the experimental design for the identification of EaRFP expressing tissue in *E. ancora* polyp (right panel). (**b–d**) Bright views of the isolated *E. ancora* tissues. (**b**) Tentacles, (**c**) Ovary, and (**d**) Mesenterial filament. (**e–g**) U-MWIG 2 (RFP) filter-fluorescent views of the same tissues shown in **b**–**d**. (**e**) Tentacles, (**f**) Ovary, and (**g**) Mesenterial filament. All bars = 500 μm. Notably, the strong red fluorescence was detected only in the ovary. (**h**) Excitation and emission spectra of the ovarian tissue extract. (**i**) Distribution of *EaRFP* transcripts in the different tissues of female polyps collected in March assessed by quantitative RT-PCR. The samples examined include isolated tentacle (Ten), ovarian tissue (Ov), and mesenterial filament (Mf). Data shown are the mean ± SE (n = 5 colonies) relative to the Ten group. Groups with different letters are significantly different (*P* < 0.05). (**j**) *EaRFP* transcript detected by Northern blotting. Molecular weight markers are shown on the left. Arrow indicates the signal.

Subsequently, we attempted to identify the tissue expressing EaRFP in the female *E. ancora* body (Fig. 2a). As a first approach, we isolated the tentacles (Fig. 2b), ovary (Fig. 2c), and the mesenterial filament (Fig. 2d) from the adult *E. ancora* collected in March (2 months before the spawning period) and observed them under a fluorescence microscope (Fig. 2e–g). Strong red fluorescence was observed only in the ovary (Fig. 2f), while the tentacles and the mesenterial filament, but not the ovary, exhibited the GFP and cyan-FP (CFP) (Supplementary Fig. 2). Spectroscopy analysis revealed that the ovarian protein extract had two major excitation peaks of 506 and 572 nm, and an emission_max_ of 594 nm with two small shoulders at 518 and 674 nm (Fig. 2h). The major spectrum observed in the ovarian protein extract was in agreement with that of recombinant EaRFP (Fig. 1e). To confirm that *EaRFP* transcripts are expressed in the ovary, we performed a tissue distribution analysis of the transcript. Significantly, high levels of *EaRFP* transcripts were detected in the ovary (Fig. 2i). Minimal levels of *EaRFP* transcripts were also detected in the tentacles, mesenterial filaments (Fig. 2i), and the male tissues (tentacles, testes, and mesenterial filaments) (data not shown). The presence of the *EaRFP* transcript was further demonstrated in the ovary by Northern blotting. A band of approximately 1.1 kb (Fig. 2j), in agreement with the size of the full-length *EaRFP* cDNA (1,087 bp), was revealed.

### Identification of the EaRFP expressing cells in the ovary

**Figure 3:**
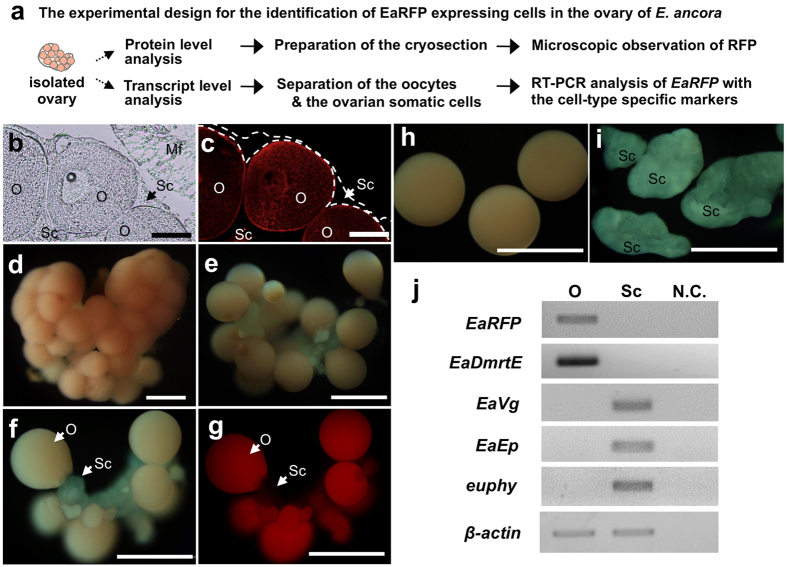
Identification of EaRFP-expressing cells in the ovarian tissue. (**a**) The experimental design for the identification of EaRFP expressing cells in the ovarian tissue at both protein and transcript levels. (**b,c**) Micrograph of cryosection of isolated ovarian tissue. (**b**) Bright view. (**c**) U-MWIG 2 (RFP) filter-fluorescent view of the same field as **b**. Broken line indicates the ovarian somatic cell layer. Red fluorescence was detected in the oocytes but not in the somatic cells. (**d–g**) Dissociation of ovarian tissues and separation of oocytes and ovarian somatic cells. (**d**) Bright view of an isolated intact ovary. (**e**) Bright view of the partially dissociated ovary by collagenase P treatment for 1 h. (**f**) Bright view of partially dissociated ovary by collagenase P treatment for 2 h. (**g**) U-MWIG 2 (RFP) filter-fluorescent view of the same field as (**f**). (**h**) Isolated oocytes. (**i**) Isolated ovarian somatic cell clumps. O, oocyte; Sc, somatic cells; Mf, mesenterial filament. Scale bar: (**b**,**c**), 100 μm; (**d**–**i**), 500μm. (**i**) Expression of *EaRFP* mRNA assessed by RT-PCR analysis. cDNA from the isolated oocytes (O) and the isolated ovarian somatic cell clumps (Sc) were used. As a marker of the oocyte, *E. ancora doublesex and mab-3 related transcription factor E* (*EaDmrtE*) were used. As the three markers of ovarian somatic cells, *E. ancora vitellogenin* (*EaVg*), *E. ancora egg protein* (*EaEp*), and a novel yolk protein in *E. ancora* (*euphy*) were used. *β-actin* was used as an internal control for RT-PCR analysis. One of the representative results of three independent experiments is shown.

Next, we attempted to determine the cell type expressing EaRFP within the ovary (Fig. 3a). A frozen section of ovarian tissue showed that EaRFP was present in the oocytes but not in the somatic cell layer of the ovarian tissue (Fig. 3b,c). To further confirm the EaRFP-expressing cell types within the *E. ancora* ovary at a transcript level, a method to separate oocytes and ovarian somatic cells was developed (Fig. 3d–i). Through the course of protease screening for dissociation, we found that collagenase P treatment could incompletely dissociate the isolated ovarian tissue (Fig. 3d,e). Fluorescent microscope observation during the ovarian dissociation also showed that EaRFP was present in the oocytes but not in the ovarian somatic cells (Fig. 3f,g). We were able to isolate the oocytes and ovarian somatic cell clumps by carefully separating them under a microscope (Fig. 3h,i). To validate the separation method, we performed a cell-type identification by RT-PCR using a molecular marker for the *E. ancora* oocyte (*E. ancora doublesex and mab-3 related transcription factor E* (*EaDmrtE*)^19^ and three molecular markers for the ovarian somatic cells of *E. ancora* (*E. ancora Vitellogenin* (*EaVg*); *E. ancora egg protein* (*EaEp*); and *euphy*)^12^,^15^. The results demonstrated that the oocytes (*EaDmrtE* positive) and the ovarian somatic cells (*EaVg*, *EaEp*, and *euphy* triple positive) were successfully separated (Fig. 3j). Subsequent RT-PCR clearly showed that *EaRFP* transcripts are present in the oocytes but not in the ovarian somatic cells (Fig. 3j), demonstrating that oocytes are endogenously expressing EaRFP in the *E. ancora* ovary.

### Determination of the oocyte stage expressing EaRFP in oogenesis

**Figure 4:**
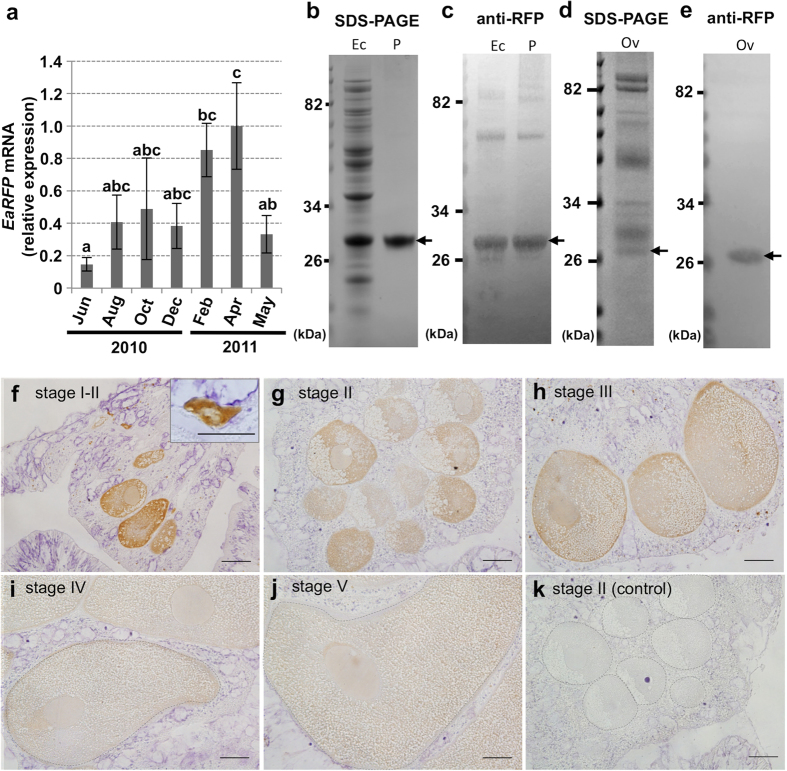
Determination of the oocyte stage that starts to express EaRFP. (**a**) Seasonal variation of *EaRFP* expression levels during 2010–2011 in female *E. ancora* polyp tissues, as assessed by quantitative RT-PCR. Data shown are the mean ± SE (n = 3 colonies) relative to the April 2011 group. Groups with different letters are significantly different (*P* < 0.05). (**b**–**e**). SDS-PAGE and Western blotting of EaRFP. (**b**) SDS-PAGE of protein extract from pET-19b *E. coli* expressing recombinant EaRFP (Ec) and purified recombinant EaRFP (P). (**c**) Western blotting of the same protein shown in (**b**) with an anti-RFP antibody. (**d**) SDS-PAGE of protein extracts of ovary (Ov) collected in March. Arrow indicates the predicted EaRFP band. (**e**) Western blotting of ovarian protein extract with an anti-EaRFP antibody. Arrow indicates an EaRFP immunoreactive band of approximately 27 kDa. Molecular weight markers are shown on the left. (**f–j**) The immunohistochemical analysis of EaRFP with an anti-RFP antibody in stage I–V oocytes. Inset in (**f**) shows the stage I oocyte. (**k**) The negative control of immunohistochemical analysis. Immunohistochemistry was performed with the omission of the 1^st^ antibody for this section (stage II oocytes). Oogenic stages I–V were classified according to cell size and morphology 6. Broken lines in (**i–k**) indicate the oocytes. All bars = 50 μm.

We subsequently addressed when the *E. ancora* oocyte starts to express EaRFP during oogenesis. Our previous study revealed that *E. ancora* has an annual reproductive cycle, and its oogenesis takes approximately 11–12 months in southern Taiwan^6^. The oogenesis usually starts in June, and the matured oocytes are released the following May^6^. Quantitative RT-PCR of cDNA from the polyp tissue samples collected in different months showed that EaRFP transcripts were already present in June-August (11–9 months before spawning), increased as April approached, and decreased somewhat during the spawning month (May) (Fig. 4a). Microscopic observation of the the ovaries collected in August, December, and March (9, 5, and 2 months before spawning, respectively) also showed that the oocytes in all the samples express EaRFP (Supplementary Fig. 3a–f).

Subsequently, an immunohistochemical analysis was performed on the oocytes at different stages (stages I–V) with a commercially available anti-RFP polyclonal antibody. The anti-RFP antibody exhibited an immunoreactivity to the recombinant EaRFP as assessed by SDS page and Western blotting (Fig. 4b,c), demonstrating that the antibody could be used for the detection of EaRFP. In addition, Western blotting of the ovarian protein showed a single immunoreactive band of approximately 27 kDa, in agreement with the predicted size of EaRFP cDNA (25.5 kDa) (Fig. 4d,e). The densitometries of the Western blotting patterns estimated that EaRFP contributes approximately 0.25% of the total protein of the *E. ancora* ovary. Subsequent immunohistochemical analysis revealed that EaRFP was present from the early to the mature stages of the oocytes (stage I–V, Fig. 4f–j). Strong immunoreactivity was observed in the oocyte cytoplasm of the early oocytes (stage I, 20–50 μm in oocyte diameters, with deformed shapes, Fig. 4f), whereas relatively weaker immunoreactivity was detected in the developing oocytes (stage II–III, 51–200 μm in oocyte diameters, Fig. 4g,h) and the mature oocytes (stage IV–V, >201 μm in oocyte diameters, Fig. 4i,j). The control section that omitted the 1^st^ antibody reactions in the procedure of immunohistochemistry exhibited no immunoreactivity (Fig. 4k). The oogonia also exhibited no immunoreactivity.

### Hydrogen peroxide degradation activity of EaRFP

**Figure 5:**
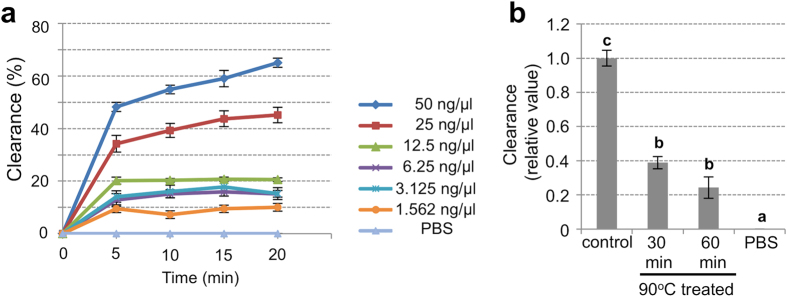
Antioxidant activity of recombinant EaRFP. (**a**) *In vitro* H_2_O_2_ degradation activity (antioxidant activity) of recombinant EaRFP. The clearance rate against protein concentration (ng/μl) and reaction time (min) was monitored to evaluate the H_2_O_2_ degradation activity of EaRFP. (**b**) H_2_O_2_ degradation activity of EaRFP (50 ng/μl) with heat-treatment (90 °C) for 30 or 60 min. Data shown are the mean ± SE relative to the intact 50 ng/μl of EaRFP (control) group (reaction time: 20 min). The assay was performed three times in quadruplicate. Groups with different letters are significantly different (*P* < 0.05).

Previous studies reported that coral FPs possess H_2_O_2_ degradation activity^20^,^21^. We also found that the recombinant EaRFP (rEaRFP) apparently exhibited an H_2_O_2_ degradation activity (Fig. 5a). At 1.56 to 6.25 ng/μl of rEaRFP, the clearance rate was slightly higher than that of the PBS (control) group. When the rEaRFP concentration reached 12.5, 25, and 50 ng ng/μl, the clearance rates were markedly increased in a dose-dependent manner (Fig. 5a). On the other hand, the clearance rates were significantly decreased in the heat-inactivated rEaRFP (Fig. 5b).

## Discussion

Stony corals have the ability to produce diversely colored FPs, such as cyan, green, orange, and red, in various tissues^22^,^23^. FPs account for a significant quantity of the soluble protein of coral tissue^24^. Distinct tissue and cellular distribution patterns of FPs within a polyp tissue (e.g., endodermal cells, ectodermal cells, tentacle, and mesenterial filament) have been reported in some corals^25^,^26^,^27^. To date, in germline cells, FPs have also been microscopically observed in the released eggs of 2 coral species, *Pocillopora verrucosa* (CFP-GFP)^16^ and *Montipora capitata* (GFP)^17^,^18^. However, the molecular identification and detailed characterization of the FPs expressed in the eggs have not been reported, and the expression of FPs during oogenesis remains unexplored. In the present study, by using an SSH screening to explore oogenesis-related genes in *E. ancora*, we identified a gene encoding a novel RFP that is highly expressed in the reproductive season. Our data clearly showed that EaRFP is almost exclusively expressed in the oocytes in the adult *E. ancora* and that EaRFP expression begins from the early stage of the oocytes (20–50 μm in diameter) and continues until the maturation period. Furthermore, EaRFP was shown to possess H_2_O_2_ degradation activity, which is in agreement with previous reports^20^,^21^. These results raise the possibility that EaRFP plays a role in protecting the oocytes from oxidative stress from the early stage of oogenesis. To the best of our knowledge, this is the first systematic study of FP using SSH-based molecular identification and spectroscopic characterization to show the spatiotemporal expression pattern in a specific cell type and to suggest a possible function. This study provides not only the first evidence for the potential involvement of FPs in oogenesis in corals but also an insight into a cellular strategy underlying coral sexual reproduction.

The previous studies showed the presence of FPs in the released eggs of two coral species that belong to different taxonomic families, *P. verrucosa* (family Pocilloporidae; CFP-GFP)^16^ and *M. capitata* (family Acroporidae; GFP)^17^,^18^. The present study demonstrated the endogenous expression of RFP in the oocytes of *E. ancora* (family Euphyllidae). In a previous study, we confirmed that RFP is also present in the oocytes of the coral *Galaxea fascicularis* (family Oculinidae) (Shikina and Chang, unpublished data). Thus, although the color of FPs in the oocytes differs among families, the previous and present studies imply that FPs may be widely observed in female germline cells in various corals across families and are potentially involved in coral oogenesis. Because the endogenous expression of GFP has also been reported in the oocytes of jellyfish *Clytia hemisphaerica*^28^, FPs may be observed in oocytes of other cnidarians.

A variety of studies have been performed on many corals to explore the function of FPs, however, their exact function remains obscure^29^. The hypothesized function of FPs include photoprotection^30^,^31^, the photosynthetic enhancement of the symbiotic algae *Symbiodinium*^30^, antioxidant capacity^20^,^21^, innate immune response^32^,^33^, and camouflage^34^. In the adult *E. ancora*, however, it is unlikely that EaRFP in the oocytes is involved in the photoprotection and symbiosis with *Symbiodinium* in the adult body because: 1) in the wild, *E. ancora* colonies inhabit sea depths of 10–15 m with no strong irradiation, and the ovarian regions are usually located inside of the polyps which are behind the skeleton with little direct sunlight irradiation; and 2) only a few *Symbiodinium* are present in the ovarian region throughout oogenesis, and to date, no *Symbiodinium* has been observed in the released eggs of *E. ancora*. In the present study, it was shown that rEaRFP has an antioxidant capacity even at low concentrations. Because coral oocytes have been shown to contain yolk proteins and lipids in their cytoplasm^10^,^11^,^12^,^15^,^18^,^35^,^36^, it is possible that EaRFP may protect the oocyte DNA and these accumulated yolk materials in the oocytes from oxidative stress starting in the early stages of oogenesis in the adult *E. ancora.* In addition, our microscopic observation revealed that the released eggs, planulae, and the larvae after settlement and metamorphosis, exhibit strong red fluorescence in *E. ancora* (Supplementary Fig. 4), suggesting that EaRFP are also present until the late larval stage. In the wild, because *E. ancora* larvae drift at the sea surface with strong irradiation, EaRFP may act as a photoprotectant. In addition, because *E. ancora* larvae obtain symbiotic algae during embryogenesis, EaRFP may also have functions related to symbiosis. It is also possible that EaRFP has other functions as described above. Currently, it is largely unclear why only the oocytes highly express “red”-FP in the adult *E. ancora*. In the future, the detailed functions of EaRFP in oogenesis and embryonic development, including other physiological roles, could be clarified by a loss of function technique, such as RNA interference.

The purified recombinant EaRFP exhibited a single excitation peak and emission maxima at 572 and 594 nm, respectively. On the other hand, the fluorescence spectrum of the ovarian protein extract exhibited multiple peaks. These results suggest that although EaRFP is one of the major proteins responsible for the endogenous red fluorescence observed in the oocytes, other types of FPs, including the non-fluorescent chromoproteins^23^, may be present in the ovary. Further molecular investigation, such as transcriptome analysis, would reveal the detailed color panel of the FPs expressing in the *E. ancora* ovary.

It was also demonstrated that EaRFP expression in the oocytes began from the early stage of oogenesis. To the best of our knowledge, this is the first study to clearly demonstrate the presence of FPs in the early oocytes among corals, and more generally cnidarians. Notably, strong immunoreactivity of EaRFP was observed in the early oocytes, whereas relatively weaker immunoreactivity was detected in the developing oocytes and the mature oocytes. This is probably caused by the dilution of EaRFP with other various proteins accumulating within the oocyte cytoplasm. Indeed, *E. ancora* oocytes have been shown to accumulate yolk proteins mainly from middle to late stage (stage II–V) of oogenesis^12^,^15^.

We previously provided the first molecular evidence for cnidarians that coral ovarian somatic cells produce three yolk proteins and supply them with oocytes^12^,^15^. This clearly indicates that ovarian somatic cells act as supporting cells for oocyte development and play key roles in oogenesis in corals. In the present study, we succeeded in establishing a series of techniques for the separation of oocytes and ovarian somatic cells. RT-PCR analysis with cell-type-specific marker genes also demonstrated the successful separation of the oocytes and ovarian somatic cells. This technique, combined with other molecular biological techniques, would allow us to perform further analyses on ovarian somatic cells at molecular levels in the future. For instance, the separated somatic cells and oocytes could be employed as materials for microarray and/or RNA-Seq analyses. This would allow us to gain a deeper understanding of the intrinsic mechanism underlying oogenesis in corals, such as the interaction between somatic cells and oocytes, including hormonal control regulated by the somatic cells.

Because the cDNA sequence and the spectroscopic character of EaRFP were revealed, EaRFP could be utilized as a tool for cell or tissue-specific labeling by transgenic technologies. Furthermore, as EaRFP is almost exclusively expressed in the oocytes in adult corals, it could be used as a suitable biomarker for oocytes, at least in *E. ancor*a. This would represent a new technique for the identification of oocytes within coral bodies. For example, as shown in Supplementary Fig. 3, RFP in the oocytes allowed us to easily confirm the presence of oocytes by observing the ovarian region under a fluorescence microscope. This method would facilitate ecological studies, such as investigations of the sexuality and maturity of coral collected from nature, in which histological analysis has been traditionally utilized to identify oocytes, especially in the samples collected during non-reproductive periods. However, because the types of FPs present in oocytes may vary among species, the species-specific expression pattern of FPs in the oocytes needs to be established for each species in the future.

## Methods

The major experimental objectives and the methods used in the present study are listed in the Supplementary Table S1.

### Experimental animal

We selected *E. ancora* as an experimental animal because: 1) they are gonochoric species and the site of gametogenesis and the annual gametogenic cycle have been revealed anatomically and histologically in both sexes^6^; and 2) they have large polyps that enable us to isolate different parts of the polyp tissue (e.g., tentacles, mesenterial filaments, and gonads) under a stereomicroscope. These attributes allowed a better analysis of mRNA and protein tissue distribution^12^.

### Coral collection and tissue isolation

Large *E. ancora* colonies were labeled and collected at different times by scuba divers over the course of several years at Nanwan Bay in Taiwan (21°57′N, 120°46′E)^6^. The collection was approved by the administration office of the Kenting National Park. For the analysis on the expression levels of mRNA at different periods during the annual reproductive cycle, a portion of the female mesenterial tissues encompassing mesenterial filaments and the ovarian tissues between two septa were collected at different times. For the analysis of mRNA and protein tissue distribution, different polyp tissues were isolated under a stereomicroscope (SZX16; Olympus, Tokyo, Japan) from the *E. ancora* collected in March. The collected samples were immediately flash frozen with liquid nitrogen and kept at −80 °C before use. The isolated tissues were observed under a fluorescence microscope (IX71SF1 equipped with U-MWU2, U-MWIB2 and U-MWIG2, Olympus). The released eggs and embryos were obtained according to the methodology described elsewhere^15^. The experiments were performed in accordance with the principles and procedures approved by the Institutional Animal Care and Use Committee, National Taiwan Ocean University, Taiwan.

### RNA and protein extraction, cDNA synthesis

The total RNAs and proteins were extracted with TRIzol reagent (Invitrogen, Carlsbad, USA) following the manufacturer’s protocol. First-strand cDNA was synthesized from 2 μg of DNase-treated RNA using Super Script II reverse transcriptase (Invitrogen).

### Construction of Suppression Subtractive Hybridization (SSH) library and screening

A differential screen by SSH was performed using the mRNA extracted from the female corals of both the non-reproductive period (June, 2010) and the reproductive period (April, 2011). Construction and screening of an SSH cDNA library were performed according to the previously described methodology^15^.

### Quantitative reverse transcription (RT)-PCR

The expression levels of *EaRFP* transcripts were analyzed by quantitative RT-PCR analysis according to the previously described methodology^15^. PCR were performed with a Bio-Rad iQ5 (Bio-Rad Laboratories, Hercules, USA) using a SYBR green dye-based assay (Bio-Rad Laboratories), according to the manufacturer’s instructions. The thermal cycling conditions were as follows: one cycle of 95 °C for 5 min, then 40 cycles of 95 °C for 15 sec, and 60 °C for 1 min. As a reference gene, *E. ancora β-actin* was used. The data were analyzed with Bio-Rad iQ5 Manager (Bio-Rad Laboratories) according to the 2^−ΔΔCt^ method^37^. Specific primers were designed using Primer Express 3.0 software (Applied Biosystems, California, USA). The primer sequences were listed in Supplementary Table S2.

### cDNA cloning, sequence and phylogenetic analysis

The full-length cDNA of clone *#2-6* (*EaRFP*) was obtained by a rapid amplification of cDNA ends (RACE) kit (SMART RACE cDNA; BD Biosciences Clontech, Franklin Lakes, USA). Touchdown PCR protocol was utilized following the manufacturer’s protocol. The primer sequences used are listed in Supplementary Table S2. The nested PCR products were ligated into the pGEM-T Easy vector (Promega, Madison, USA), transformed into competent cells (*Escherichia coli*, JM109 strain, Promega), and sequenced using an ABI Prism 310 Genetic Analyzer (Applied Biosystems). The molecular weight was predicted using the Compute pI/Mw tool (http://web.expasy.org/compute_pi/). For the phylogeny analysis, a subset of the RFP subfamily members from different species was retrieved from GenBank and phylogenetically compared with EaRFP obtained here. Alignments were performed using MUSCLE, and the results were used to create a 10,000 bootstrap replicate phylogenetic tree (neighbor-joining method) using MEGA 6^38^.

### Transfection and HEK293 cell culture

The ORF of *EaRFP* cDNA was ligated into the expression vector pCDNA3.1 (+) (Invitrogen). The pCDNA3.1 (+)-*EaRFP* plasmid or empty pCDNA3.1 (+) vector (negative control) were transiently transfected into the HEK293 cell line using Lipofectamine 3000 (Invitrogen) according to the manufacturer’s protocol. Before the day of transfection, 0.7 × 10^5^ cells were seeded onto a 12-well culture plate and grown in Dulbecco’s modified Eagle’s medium (DMEM, Invitrogen) supplemented with 10% fetal bovine serum (Invitrogen), 2 mM sodium pyruvate (Sigma-Aldrich, St Louis, USA) and antibiotics (50 μg/ml ampicillin, 50 U/ml penicillin, and 50 μg/ml streptomycin; Invitrogen) at 37 °C under 5% CO_2_ atmosphere. The cells were transfected with 1.25 μg of each plasmid in 1 ml Opti-MEM (Invitrogen).

### Bacterial culture, induction and purification of recombinant EaRFP

ORF of *EaRFP* cDNA were inserted into pET-19b vector (Novagen Merck KGaA, Darmstadt, Germany). *E. coli* BL21 (DE3) pLysS competent cells (Novagen) were used as a host strain for the recombinant construct and grown at 37 °C until reaching log phase. The expression of the fusion protein was then induced by the addition of 1 mM of isopropyl-β-D-thiogalactopyranoside, followed by culture at 28 °C for 16 h in LB medium. N-terminal His-tagged recombinant EaRFP was purified from the cell lysate by passing through an affinity column containing a high density of nickel-agarose beads (Agarose Bead Technologies, Madrid, Spain).

### Spectroscopy

The excitation and emission of both purified recombinant EaRFP and ovarian extract protein were measured using Synergy^TM^ MxMonochromator-based multi-mode microplate reader (BioTek Instruments, Inc., Vermont, USA). To determine the excitation and emission maxima, a white laser was used to excite with a continuous spectrum, including all wavelengths from blue to infrared. The ovarian extracts were prepared by homogenizing the ovaries in phosphate-buffered saline (PBS) with a sonicator (Sonics and Materials Inc., Newtown, USA). The homogenates were centrifuged at 13,000 rpm for 10 min at 4 °C. The supernatants were collected, filtrated with a 0.22-μm filter to remove the remaining cell debris and subjected to the spectroscopy analysis.

### Northern blotting

An RNA probe was synthesized using a 741 bp partial *EaRFP* cDNA fragment (nucleotides 50–790 of *EaRFP*). RNA probe synthesis and Northern blotting were performed according to a previously described methodology^12^.

### Frozen section

The ovaries isolated from *E. ancora* were fixed for 16 h in 20% Zinc Formal-Fixx (Thermo Shandon, Pittsburgh, USA)/0.22 μm filtered seawater. The samples were processed through 30% sucrose, embedded in optimum cutting temperature (O.C.T) compound (Sakura Finetek USA Inc., Torrance, USA), and sectioned at a thickness of 5 μm with a cryostat (CM3050S; Leica Microsystems, Wetzlar, Germany). Sections were directly observed under a fluorescence microscope (IX71SF1 equipped with U-MWIG2, Olympus).

### Dissociation of ovarian tissues and separation of oocytes and ovarian somatic cells

Ovarian tissues isolated from *E. ancora* collected in April were dissociated using 1 mg/ml collagenase P (Roche, Mannheim, Germany) in 0.22 μm filtered artificial seawater for 2 h at room temperature. During the dissociation, gentle pipetting was applied every 30 min to disperse the ovarian tissues. The oocytes and the clumps of ovarian somatic cells were isolated under a stereomicroscope.

### SDS-PAGE and Western blotting

SDS-PAGE and Western blotting were performed according to the methodology described elsewhere^12^. The 10 μg of protein extract from pET-19b *E. coli* expressing recombinant EaRFP, the purified recombinant EaRFP, or the ovarian proteins were used for the analyses. For SDS-PAGE, proteins were separated by 10% polyacrylamide gel (Invitrogen) and visualized with Coomassie blue R250 stain (Bio-Rad Laboratories). For Western blotting, the RFP antibody (Catalog No. ab124754, Abcam, Cambridge, UK) was diluted (1: 4,000) in Tris-buffered saline and 0.1% Tween 20 (TBT) with 2% skim milk and used for the 1^st^ antibody reaction. For the 2^nd^ antibody reaction, 0.25 μg/ml alkaline phosphatase-conjugated goat anti-rabbit IgG antibody (AnaSpec, San Jose, USA) diluted in TBT with 1% skim milk was used. Immunoreactive bands were visualized by the NBT/BCIP system (Sigma-Aldrich).

### Immunohistochemistry

The female *E. ancora* sampled at different times were fixed at room temperature for 16 h in 20% Zinc Formal-Fixx/0.22 μm filtered seawater. Immunohistochemistry was performed according to a previously described methodology^39^. For the 1^st^ antibody reaction, the RFP antibody (ab124754, Abcam) diluted in PBS containing 0.1% Tween 20 (PBT) (1:2,000) with 2% skim milk was used. For the 2^nd^ antibody reaction, a biotinylated goat anti-rabbit IgG antibody (Vector Laboratories, Burlingame, USA; diluted 1:2,000 with PBT, 2% BSA) was used. The immunoreactivity was visualized by 3,3′-diaminobenzidine (DAB; Sigma-Aldrich).

### H_2_O_2_ degradation assay

The H_2_O_2_ degradation activity of EaRFP was investigated according to the methodology reported by Palmer *et al.*^21^, with some modification. Briefly, 20 μl of the purified-recombinant EaRFP (rEaRFP) or the heat-inactivated rEaRFP was mixed with 30 μl of PBS in wells of a 96-well UV transparent microtiter plate (Coaster, Corning, USA). To each well, 50 μl of 50 mM H_2_O_2_ was added and the absorbance at 230 nm was read every 30 sec for 20 min by Synergy^TM^ HT microplate reader (BioTek Instruments). The final concentration of rEaRFP prepared by serial dilution in the wells ranged from 50 ng/μl to 1.56 ng/μl. Sample blanks and wells containing only PBS were used as controls. For the quantification of the H_2_O_2_ degradation activity, a standard curve was created by the absorbance from 50 mM to 3.125 mM of H_2_O_2_ on the same plate. The clearance rates were calculated using the following formula: clearance rate (%) = 100 × [(A_0_ − A*x*)/A_0_], where A_0_ was the initial absorbance and A*x* was the absorbance at each time point. The assay was performed three times in quadruplicate.

### Statistics

The data are shown as the mean ± standard error (SE). Statistical significance was determined using Student’s *t*-test for comparisons between two groups. For comparisons among more than 3 groups, Statistical Package for the Social Sciences (SPSS) software was used for the analysis of statistical significance with a one-way analysis of variance (ANOVA), followed by Tukey’s test. The statistical significance level was set to *P* < 0.05.

## Additional Information

**How to cite this article**: Shikina, S. *et al.* Oocytes express an endogenous red fluorescent protein in a stony coral, *Euphyllia ancora:* a potential involvement in coral oogenesis. *Sci. Rep.* **6**, 25868; doi: 10.1038/srep25868 (2016).

## Supplementary Material

Supplementary Information

## References

[b1] Odum H. T. & Odum E. P. Trophic structure and productivity of a windward coral reef community on Eniwetok Atoll. Ecol. Monogr. 25, 291–320 (1955).

[b2] Twan W. H. *et al.* Hormones and reproduction in scleractinian corals. Comp. Biochem. Physiol., Part A Mol. Integr. Physiol. 144, 247–253 (2006). 10.1016/j.cbpa.2006.01.011 16488637

[b3] Harrison P. L. Coral Reefs: Ecosystem in Transition. (ed. Dubinsky Z. & Stambler N. ) 59–85 (Springer, 2011).

[b4] Harrison P. L. & Wallace C. C. Ecosystems of the world 25: Coral reefs. (ed. Dubinsky Z. ) 133–207 (Elsevier, 1990).

[b5] Baird A.H., Guest J. R. & Willis B. L. Systematic and biogeographical patterns in the reproductive biology of scleractinian corals. Annu. Rev. Ecol. Evol. Syst. 40, 551–571 (2009).

[b6] Shikina S. *et al.* Germ cell development in the scleractinian coral *Euphyllia ancora* (Cnidaria, Anthozoa). PLOS ONE 7, e41569 (2012). 10.1371/journal.pone.0041569 PMC3407244 22848529

[b7] Shikina S. *et al.* Localization of early germ cells in a stony coral, *Euphyllia ancora*: potential implications for a germline stem cell system in coral gametogenesis. Coral Reefs 34, 639–653 (2015a).

[b8] Imagawa S., Nakano Y. & Watanabe T. Molecular analysis of a major soluble egg protein in the scleractinian coral *Favites chinensis*. Comp. Biochem. Physiol. B, Biochem. Mol. Biol. 137, 11–19 (2004). 10.1016/j.cbpc.2003.09.011 14698906

[b9] Hayakawa H., Nakano Y., Andoh T. & Watanabe T. Sex-dependent expression of mRNA encoding a major egg protein in the gonochoric coral *Galaxea fascicularis*. Coral Reefs 24, 488–494 (2005).

[b10] Hayakawa H., Andoh T. & Watanabe T. Precursor structure of egg proteins in the coral *Galaxea fascicularis*. Biochem. Biophys. Res. Commun. 344, 173–180 (2006). 16616007 10.1016/j.bbrc.2006.03.116

[b11] Hayakawa H., Andoh T. & Watanabe T. Identification of a novel yolk protein in the hermatypic coral *Galaxea fascicularis*. Zool. Sci. 24, 249–255 (2007). 17551245 10.2108/zsj.24.249

[b12] Shikina S. *et al.* Yolk formation in a stony coral *Euphyllia ancora* (Cnidaria, Anthozoa): insight into the evolution of vitellogenesis in non-bilaterian animals. Endocrinology 154, 3447–3459 (2013). 23766130 10.1210/en.2013-1086

[b13] Shinzato C. *et al.* Using the *Acropora digitifera* genome to understand coral responses to environmental change. Nature 476, 320–323 (2011). 21785439 10.1038/nature10249

[b14] Diatchenko L. *et al.* Suppression subtractive hybridization: a method for generating differentially regulated or tissue-specific cDNA probes and libraries. Proc. Natl. Acad. Sci. USA 93, 6025–6030 (1996). 8650213 10.1073/pnas.93.12.6025 PMC39182

[b15] Shikina S., Chiu Y. L., Lee Y. H. & Chang C. F. From somatic cells to oocytes: a novel yolk protein produced by ovarian somatic cells in a stony coral, Euphyllia ancora. Biol. Reprod. 93, 57 (2015b). 26178717 10.1095/biolreprod.115.129643

[b16] Hirose M., Kinzie R. A. & Hidaka M. Early development of zooxanthella-containing eggs of the corals *Pocillopora verrucosa* and *P. eydouxi* with special reference to the distribution of zooxanthellae. Biol. Bull. 199, 68–75 (2000). 10.2307/1542708 10975644

[b17] Roth M. S., Alamaru A., Padilla-Gramino J. L. & Gates R. D. Fluorescence in eggs of the coral *Montipora capitata*. In: The biology of corals: Developing a fundamental understanding of the coral stress response, 2007 Edwin W. Pauley Summer Program in Marine Biology (ed. Gates R. D. ) 95 (Kaneohe, 2007).

[b18] Padilla-Gamiño J. L. *et al.* Are all eggs created equal? A case study from the Hawaiian reef-building coral *Montipora capitata*. Coral Reefs 32, 137–152 (2013).

[b19] Chen C. J. *et al.* A novel female-specific and sexual reproduction associated Dmrt gene discovered from a stony coral, *Euphyllia ancora* (Cnidaria, Anthozoa). Biol. Reprod. 94, 40 (2016). 10.1095/biolreprod.115.133173 26740592

[b20] Bou-Abdallah F., Chasteen N. D. & Lesser M. P. Quenching of superoxide radicals by green fluorescent protein. Biochim. Biophys. Acta 1760, 1690–1695 (2006). 10.1016/j.bbagen.2006.08.014 PMC1764454 17023114

[b21] Palmer C. V., Modi C. K. & Mydlarz L. D. Coral fluorescent proteins as antioxidants. PLOS ONE 4, e7298 (2009). 10.1371/journal.pone.0007298 PMC2752795 19806218

[b22] Dove S. G., Hoegh-Guldberg O. & Ranganathan S. Major colour patterns of reef-building corals are due to a family of GFP-like proteins. Coral Reefs 19, 197–204 (2001).

[b23] Alieva N. O. *et al.* Diversity and evolution of coral fluorescent proteins. PLOS ONE 3, e2680 (2008). 10.1371/journal.pone.0002680 PMC2481297 18648549

[b24] Leutenegger A. *et al.* It’s cheap to be colorful: anthozoans show a slow turnover of GFP-like proteins. FEBS J. 274, 2496–2505 (2007). 10.1111/j.1742-4658.2007.05785.x 17419724

[b25] Kelmanson I. V. & Matz M. V. Molecular basis and evolutionary origins of color diversity in great star coral *Montastraea cavernosa* (Scleractinia: Faviida). Mol. Biol. Evol. 20, 1125–1133 (2003). 10.1093/molbev/msg130 12777529

[b26] Oswald F. *et al.* Contributions of host and symbiont pigments to the coloration of reef corals. FEBS J. 274, 1102–1122 (2007). 10.1111/j.1742-4658.2007.05661.x 17244197

[b27] Gruber D. F., Kao H. T., Janoschka S., Tsai J. & Pieribone V. A. Patterns of fluorescent protein expression in Scleractinian corals. Biol. Bull. 215, 143–154 (2008). 10.2307/25470695 18840775

[b28] Fourrage C., Swann K., Garcia J. R. G., Campbell A. K. & Houliston E. An endogenous green fluorescent protein-photoprotein pair in *Clytia hemisphaerica* eggs shows co-targeting to mitochondria and efficient bioluminescence energy transfer. Open Biol. 4, 130206 (2014). 10.1098/rsob.130206 PMC4043110 24718596

[b29] Roth M. S. & Deheyn D. D. Effects of cold stress and heat stress on coral fluorescence in reef-building corals. Sci. Rep. 3, 1421 (2013). 10.1038/srep01421 PMC3594756 23478289

[b30] Salih A., Larkum A., Cox G., Kühl M. & Hoegh-Guldberg O. Fluorescent pigments in corals are photoprotective. Nature 408, 850–853 (2000). 10.1038/35048564 11130722

[b31] Roth M. S., Latz M. I., Goericke R. & Dehyn D. D. Green florescent protein regulation in the coral *Acropora yongei* during photoacclimation. J. Exp. Biol. 213, 3644–3655 (2010). 10.1242/jeb.040881 20952612

[b32] Palmer C. V., Roth M. S. & Gate R. D. Red fluorescent protein responsible for pigmentation in trematode-infected *Porites compressa* tissues. Biol. Bull. 216, 68–74 (2009). 10.1086/BBLv216n1p68 19218493

[b33] D’Angelo C. *et al.* Locally accelerated growth is part of the innate immune response and repair mechanisms in reef-building corals as detected by green fluorescent protein (GFP)-like pigments. Coral Reefs 31, 1045–1056 (2012).

[b34] Matz M. V., Marshall N. J. & Vorobyev M. Symposium-in-print: green fluorescent protein and homologs. Photochem. Photobiol. 82, 345–350 (2006). 10.1562/2005-08-18-RA-653 16613484

[b35] Arai T. *et al.* Lipid composition o positively buoyant eggs of reef building corals. Coral Reefs 12, 71–75 (1993).

[b36] Lin C., Wang L. H., Meng P. J., Chen C. S. & Tsai S. Lipid content and composition of oocytes from five corals species: potential implications for future cryopreservation efforts. PLOS ONE 8, e57823 (2013). 10.1371/journal.pone.0057823 PMC3585170 23469074

[b37] Livak K. J. & Schmittgen T. D. Analysis of relative gene expression data using real-time quantitative PCR and the 2(−Delta Delta C(T)) Method. Methods 25, 402–408 (2001). 10.1006/meth.2001.1262 11846609

[b38] Tamura K., Stecher G., Peterson D., Fillipski A. & Kumar S. MEGA6: molecular evolutionary genetics analysis version 6.0. Mol. Biol. Evol. 30, 2725–2729 (2013). 10.1093/molbev/mst197 PMC3840312 24132122

[b39] Shikina S. *et al.* Molecular cloning and characterization of a steroidogenic enzyme, 17β-hydroxysteroid dehydrogenase type 14, from the stony coral *Euphyllia ancora* (Cnidaria, Anthozoa). Gen. Comp. Endocrinol. 228, 95–104 (2016). 10.1016/j.ygcen.2016.02.006 26868454

