# Supplementary material for: Oocytes express an endogenous red fluorescent protein in a stony coral, Euphyllia ancora: a potential involvement in coral oogenesis
Source: Sci Rep. 2016 May 11;6:25868. doi: 10.1038/srep25868 (PMC4863156; doi:10.1038/srep25868)
Supplement: Supplementary Information [file srep25868-s1.pdf]

Supplementary Information

Title: Oocytes express an endogenous red fluorescent protein in a stony coral, *Euphyllia ancora*: a potential involvement in coral oogenesis.

Authors: Shinya Shikina, Yi-Ling Chiu, Yi-Jou Chung, Chieh-Jhen Chen, Yan-Horn Lee, and Ching-Fong Chang

Supplementary Table S1, Table S2, Figures, and Figure Legends

Supplementary Table S1. The major experimental objectives, the methods, and the figures showing the results in the present study

**Objectives**	**Methods**	**Figures (Results)**
Identificaiton of clone #2-6 (EaRFP)	Suppression subtractive hybridization (SSH), Screening of SSH cDNA library, and Comparison of transcript levels of *#2-6* in *E. ancora* tissue between reproductive and non-reproductive periods by quantitative reverse transcription PCR (qRT-PCR)	Fig.1
Molecular characterization of clone #2-6 (EaRFP)	The full-length cloning by RACE-PCR, Phylogenetic analysis, Transient expression of #2-6 in HEK293 cells, Recombinant #2-6 expression in *E. coli*, and Spectroscopy	Fig.1 & Sup. Fig.1
Identification of EaRFP expressing tissue in the polyp	Microscopic observation of the isolated tissues, Spectroscopy, *EaRFP* mRNA tissue distribution analysis by qRT-PCR, and Northern blotting	Fig.2 & Sup. Fig.2
Identification of EaRFP expressing cells in the ovary	Cryosection of *E. ancora* ovary, Microscopic observation of the cryosection, Separation of the oocytes and the ovarian somatic cells, and RT-PCR analysis	Fig.3
Determination of the oocyte stage expressing EaRFP	qRT-PCR analysis, Microscopic observation of EaRFP in the ovarian tissue at different developmental stage of oogenesis, Antibody validation by Western blotting, and Immunohistochemical analysis on the oocytes at different stages	Fig.4 & Sup. Fig.3
Investigation of the possible function of EaRFP	Tests for the hydrogen peroxide degradation activity of recombinant EaRFP	Fig.5
Investigation of the presence of EaRFP in the embryo	Microscopic observation of EaRFP in the released eggs and the embryos	Sup. Fig.4

Supplementary Table S2. Primers used in the RT-PCR, quantitative RT-PCR, and 3’ and 5’ RACE

Primer ID	PCR type	5'-3' Sequence	T_m (^oC)	Amplicon size (bp)
EaRFP ORF F	RT-PCR	GCCAAGCGTCTTACTCCGGAT	60	741
EaRFP ORF R	RT-PCR	GCTCAGAGTGAACTGGTTCATT		
EaRFP 5' 1	5’ RACE	TGGCCACCTCCTTCCACCTTAAGGACCA	66	580
EaRFP 5' 2	5’ RACE	CAGCGTCTCCACGGATGGCTCCCATCCT	68	515
EaRFP 3' 1	3’ RACE	CCACCGACTTGAACTAACGAGCCACGACGA	67	424
EaRFP 3' 2	3’ RACE	ACAGCACGAGTTTGCAGTCGCTCGCTGT	66	376
EaRFP F1	Probe synthesis	GCCAAGCGTCTTACTCCGGAT	60	741
EaRFP R1	Probe synthesis	GCTCAGAGTGAACTGGTTCATT		
EaRFP (#2-6) F	Quantitative RT-PCR	ATCCCCTGTCTGTAAACTGCCC	60	128
EaRFP (#2-6) R	Quantitative RT-PCR	TGGGCAAAGGAGAACAGCGA		
EaRFP F2	pET19b-recombinant	CATATGATGAGTGTGATTAAA	45	694
EaRFP R2	pET19b-recombinant	GCTCAGCAGTAAGGATCCG		
EaRFP F3	HEK293-transfection	GGATCCATTATGGGTGTGA	50	696
EaRFP R3	HEK293-transfection	GAATTCTTACTGCTGAGC		
EaVg F	RT-PCR	GGAAATCACGGACCATCTCT	60	152
EaVg R	RT-PCR	GCTGGAATCTACTCTGCTTGC		
EaEp F	RT-PCR	CCCGTCGCTTGAGAGTGGC	60	103
EaEp R	RT-PCR	GGTCCATCCCCCATCTCCAC		
euphy F	RT-PCR	GGAGCAAGCGGGATACGAAAT	60	149
euphy R	RT-PCR	GTTTACTATTCCCAATGCCATGACC		
EaDmrtE F	RT-PCR	GCAACGAACAGCATGGGACTTCC	60	172
EaDmrtE R	RT-PCR	CCTGGCCACATACTTTGCACAC		
β-actin F	RT-PCR	CGCCTTCCTTGGAATGGAATCCTCT	60	151
β-actin R	RT-PCR	CTGCATCCTGTCAGCGATTCCAGG		

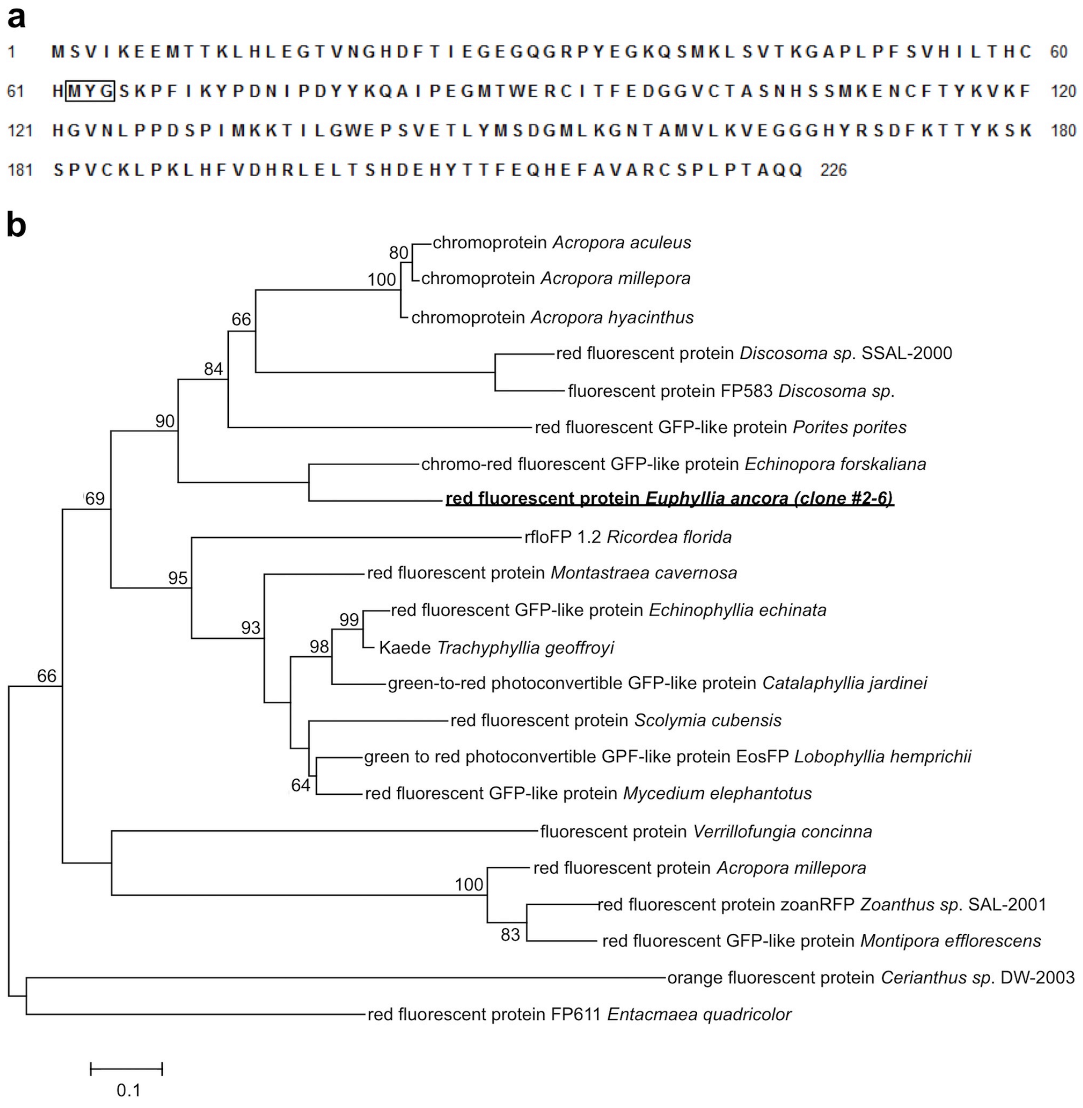

Supplementary Fig.1

Supplementary Fig. 1

Deduced amino acid sequences of clone #2-6 (EaRFP) and phylogenetic tree comparing with RFP sequences from other anthozoans in Cnidaria. (**a**) The deduced amino acid sequences of clone #2-6 (EaRFP). Black box, chromophore-forming residues. Sequence data are available from GenBank, under accession number KT452623. (**b**) A

phylogenetic tree comparing the amino acid sequences of RFP from various anthozoans. The number at each node represents the bootstrap probability (%); the branches shown correspond to values of 50 % and higher. The corresponding GenBank accession numbers of the proteins analyzed are as follows: *Acropora aculeus* (AAU06856), *A. millepora* (AAU06854), *A. hyacinthus* (AAU06855), *Discosoma* sp. SSAL-2000 (AAG16224), *Discosoma* sp. (AAF03369), *Porites porites* (ABB17953), *Echinopora forskaliana* (ACD13196), *Euphyllia ancora* (KT452623), *Ricordea florida* (AAK71339), *Montastraea cavernosa* (AAO61598), *Echinophyllia echinata* (ABB17960), *Trachyphyllia geoffroyi* (BAC20344), *Catalaphyllia jardinei* (ABN41777), *Scolymia cubensis* (AAU06843), *Lobophyllia hemprichii* (AAV54099), *Mycedium elephantotus* (ABB17959), *Verrillofungia concinn* (BAD24721), *A. millepora* (AAU06852), *Zoanthus* sp. (AAL23574), *Montipora efflorescens* (ABB17952), *Cerianthus* sp. (AAP55761), *Entacmaea quadricolor* (AAN05449).

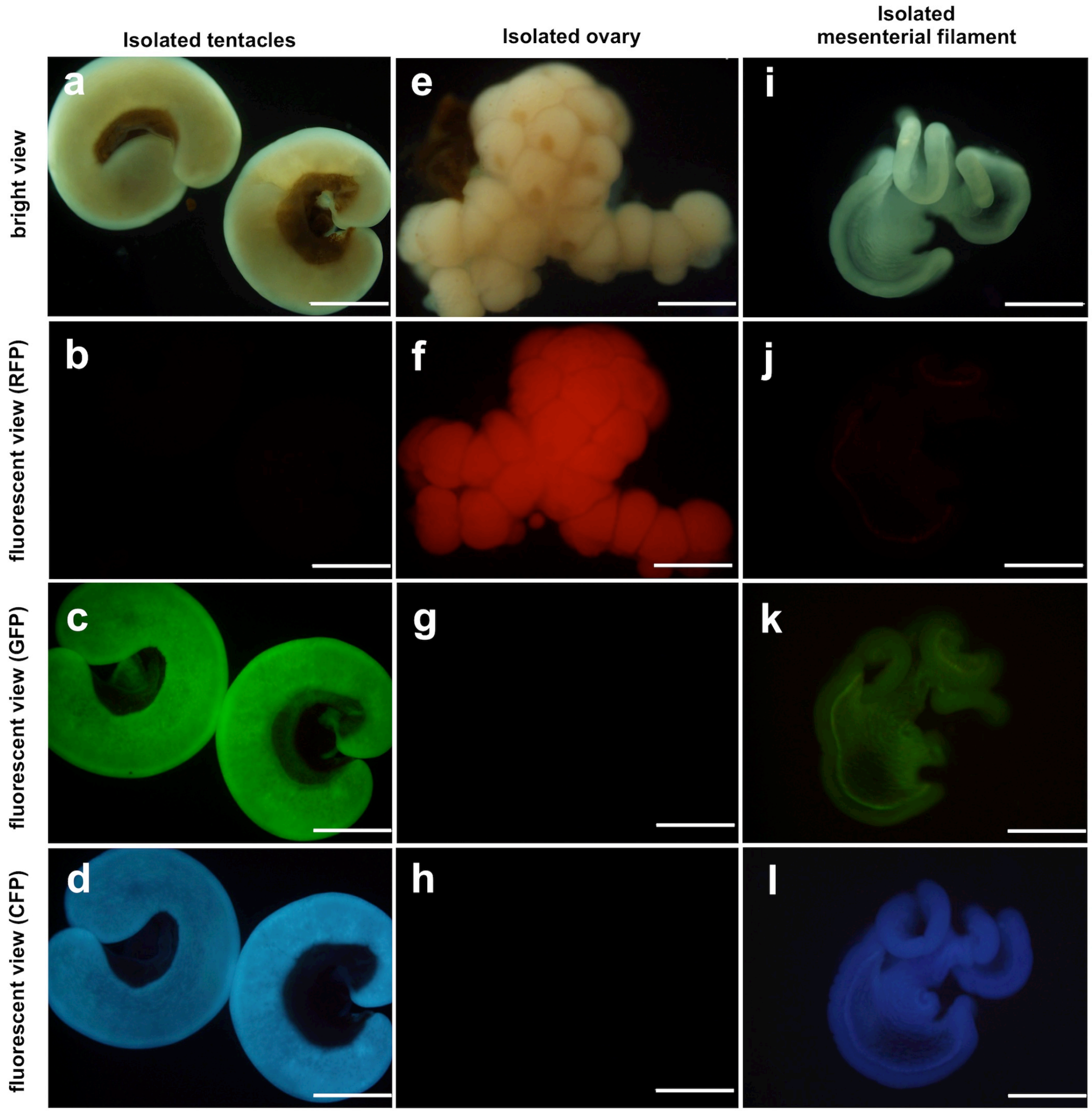

Supplementary Fig. 2

Supplementary Fig. 2

Fluorescence microscopic observation of the isolated *E. ancora* tissues. a-d) Isolated tentacles (Ten), **e-h**) Isolated ovary (Ov), **i-l**) Isolated mesenterial filament (Mf). **a**, **e**, and **i**) Bright view. **b**, **f**, and **j**) U-MWIG 2 (RFP) filter view. **c**, **g**, and **k**) U-MWIB 2 (GFP) filter view. **d**, **h**, and **l**) U-MWU2 (CFP) filter view. All bars = 500 µm.

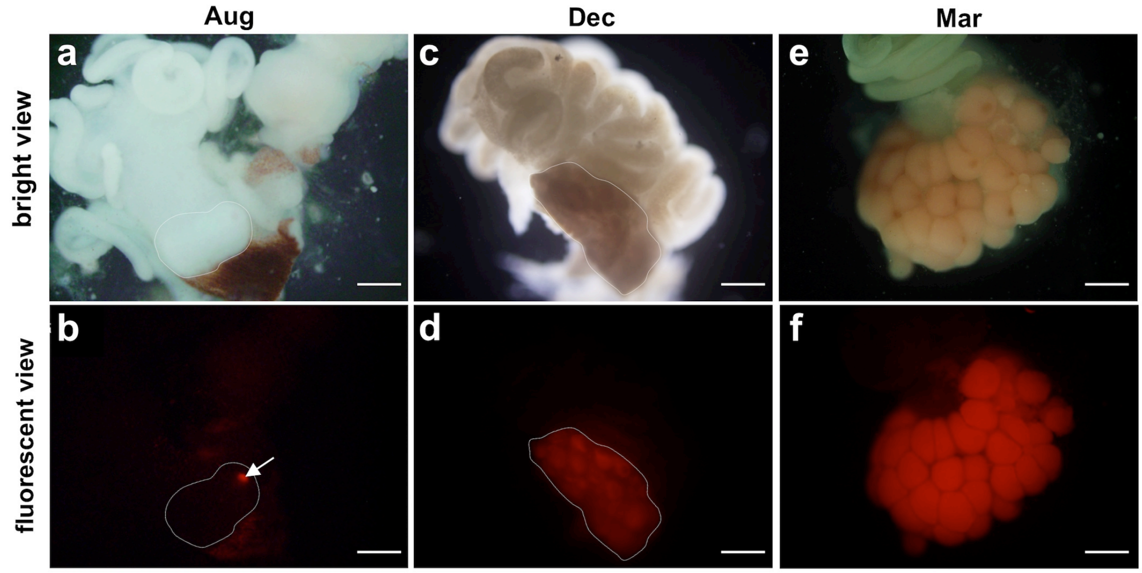

Supplementary Fig.3

Supplementary Fig. 3

Fluorescence microscopic observation of EaRFP in the ovarian tissues with mesenterial filaments. (**a**,**b**) The sample collected in August (Aug). (**c**,**d**) The sample collected in December (Dec). (**e**,**f**) The sample collected in March (Mar). **a**, **c**, and **e**: bright views; **b**, **d**, and **f**: U-MWIG 2 (RFP) filter-fluorescent views. All bars = 500 μm.

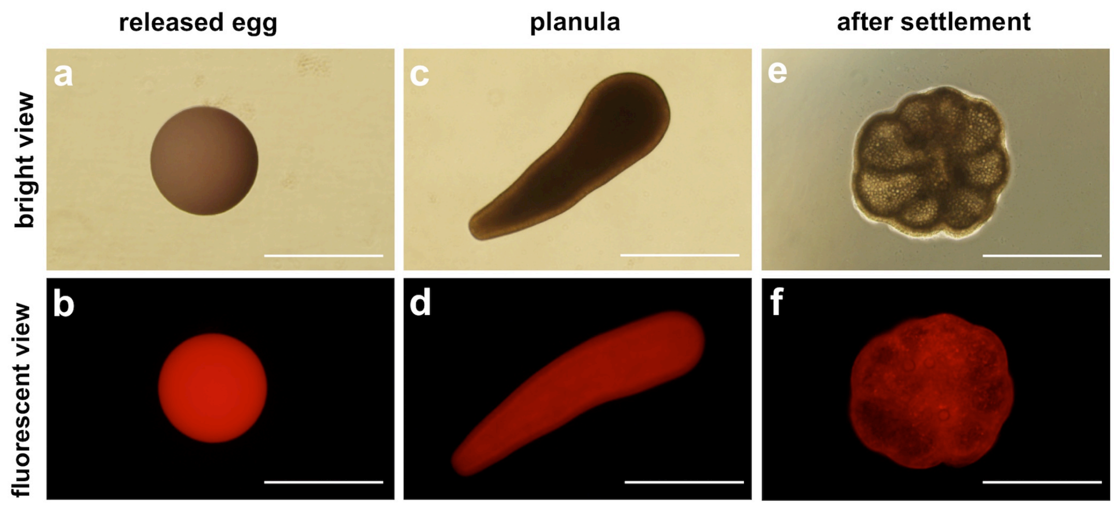

Supplementary Fig. 4

Supplementary Fig. 4

Fluorescence microscopic observation of EaRFP in the released egg and the embryos. (**a,b**) The released egg. (**c,d**) The planula larva (**e,f**) The larva after settlement and metamorphosis. **a**, **c**, and **e**: bright views; **b**, **d**, and **f**: U-MWIG 2 (RFP) filter-fluorescent views. All bars = 500 µm.
